# Abducens nerve palsy in patients with spontaneous intraventricular hemorrhage: A case report

**DOI:** 10.1097/MD.0000000000041447

**Published:** 2025-02-14

**Authors:** Chenyan Li, Yong Cai, Xingming Zhong, Lijuan Shen

**Affiliations:** a HuZhou University, HuZhou, Zhejiang, China; b The Department of Neurosurgery, The First People’s Hospital of Huzhou, Zhejiang, China.

**Keywords:** abducens nerve palsy, case report, digital subtraction angiography, intracranial hypertension, spontaneous ventricular hemorrhage

## Abstract

**Rationale:**

The purpose of this case report is to describe the process of the patient developing abductor nerve paralysis, analyze the causes of this symptom, provide new diagnostic and treatment ideas for patients with spontaneous intraventricular hemorrhage who develop abductor nerve paralysis, and be alert to the occurrence of abductor nerve paralysis in such patients.

**Patient concerns:**

A 49-year-old man was admitted to our hospital with a spontaneous intraventricular hemorrhage. After performing digital subtraction angiography of the whole brain under local anesthesia, his eyes were limited in abduction, accompanied by hemorrhage and exudation of the optic papilla. Edema is an extremely rare condition.

**Diagnoses:**

Transient intracranial hypertension was considered after reexamination with computed tomography.

**Interventions:**

The application of mannitol and the release of cerebrospinal fluid were used to reduce intracranial pressure and provide patient-related symptomatic treatment.

**Outcomes:**

After 1 month, the patient’s eyes had no abduction limitation and showed good activity.

**Lessons:**

Abducens nerve palsy is a common condition. Owing to its complex etiology, a careful and comprehensive examination is necessary. In particular, patients with spontaneous cerebral hemorrhage who undergo digital subtraction angiography should pay more attention to the abducens nerve.

## 1. Introduction

Spontaneous intracerebral hemorrhage refers to cerebral parenchymal hemorrhage caused by the spontaneous rupture of large and small intracranial arteries, veins, and capillaries in adults, which is not caused by trauma.^[1]^ Although the incidence rate of spontaneous cerebral hemorrhage only accounts for 10% to 15% of all types of stroke,^[2]^ the mortality rate accounts for 50% to 70% of all types of stroke.^[3]^ It is a destructive neurological disease with high disability and mortality rates.^[4,5]^ Patients face a high risk of neurological deterioration in the early stages of the disease,^[6]^ exhibiting various symptoms such as motor disorders, sensory abnormalities, and unclear speech, which seriously affect their daily living abilities and lead to a sharp decline in their quality of life,^[7]^ causing a heavy psychological burden and economic pressure on patients and their families. Simultaneously, it exacerbates the deterioration of patients’ neurological function, prolongs hospitalization time, and increases the mortality and disability rates of patients.^[8]^

The abductor nerve is the sixth cranial nerve, a motor nerve that innervates the ipsilateral lateral rectus muscle to control eyeball abduction.^[9]^ The abductor nerve originates from the abductor nerve nucleus in the tegmental area of the pons, exits the brain from the brainstem sulcus of the medulla oblongata, runs obliquely outward and upward, passes through the dura mater below the posterior clinoid process, enters the Dorello tubule, and travels inside the cavernous sinus.^[10,11]^ It travels along the inferior side of the internal carotid artery, enters the orbit through the supraorbital fissure, and is distributed throughout the lateral rectus muscle. By controlling contraction and relaxation, it regulates the horizontal outward or inward rotation of the eyeball.^[12]^

The incidence of abducens nerve palsy (ANP) is 11.3/100,000.^[13]^ ANP is caused by paralysis or limited movement of 1 or more extraocular muscle. It is usually seen in 1 or 2 cases of external rectus paralysis or bilateral abducens nerve paralysis.^[14]^

ANP can be classified into nuclear, fascicular, and peripheral lesions.^[10]^ It is a disease with limited ocular abduction and diplopia caused by a variety of reasons and may occasionally cause dizziness, headache, and other clinical manifestations.^[15]^ It is the most commonly acquired ocular motor nerve palsy.^[16,17]^ Most patients with diplopia seriously affect their normal work and life, and often like to cover or close their eyes.^[18]^ The causes of ANP are complex and diverse and include hypertension, diabetes, trauma, cerebrovascular disease, space-occupying lesions, and nonspecific inflammation.^[19]^ However, it is difficult to directly cause spontaneous ventricular hemorrhage, both anatomically and pathophysiologically. In February 2021, our department admitted a rare case of ANP caused by spontaneous intraventricular hemorrhage. The remainder of this paper is as organized.

## 2. Case presentation

A 49-year-old male presented with a sudden headache without obvious inducement half a day prior. He was distending, not dramatic, and tolerable, with nausea and vomiting several times. The vomiting was gastric content, non-ejection, diarrhea several times, loose stool, no improvement after rest, no chills and fever, no blurred vision, no chest tightness, chest pain, no limb weakness, convulsions, and other discomfort, and was then sent to the emergency department of our hospital. The patient has been mentally confused and weak, with poor appetite and average sleep since falling ill. Recently there has been no significant increase or decrease in body weight. The patient reported that he had a medical history of “diabetes” for more than 1 year, and regularly took the hypoglycemic drugs metformin hydrochloride sustained-release tablets and glimepiride tablets, but the blood glucose control was general.

## 3. Physical examination

On admission, the blood pressure was 190/134 mm Hg, heart rate was 109 times/min, body temperature was 36 °C, respiration was 20 times/ min, blood oxygen saturation was 98 %, BMI was 33.41, consciousness was blurred, passive position, and acute appearance. The pain score was 2 points (using a numerical rating scale). Physical examination upon admission revealed unclear consciousness, GCS13 score, bilateral pupils of equal size and diameter (2.5 mm, slow light reflex), normal muscle tone in limbs, limb muscle strength grade V, and negative bilateral Babbitt sign. The rhythm was regular, without obvious pathological murmurs, lung breathing sounds were coarse, and there were no obvious dry wet rales.

## 4. Diagnosis

**Figure 1.**
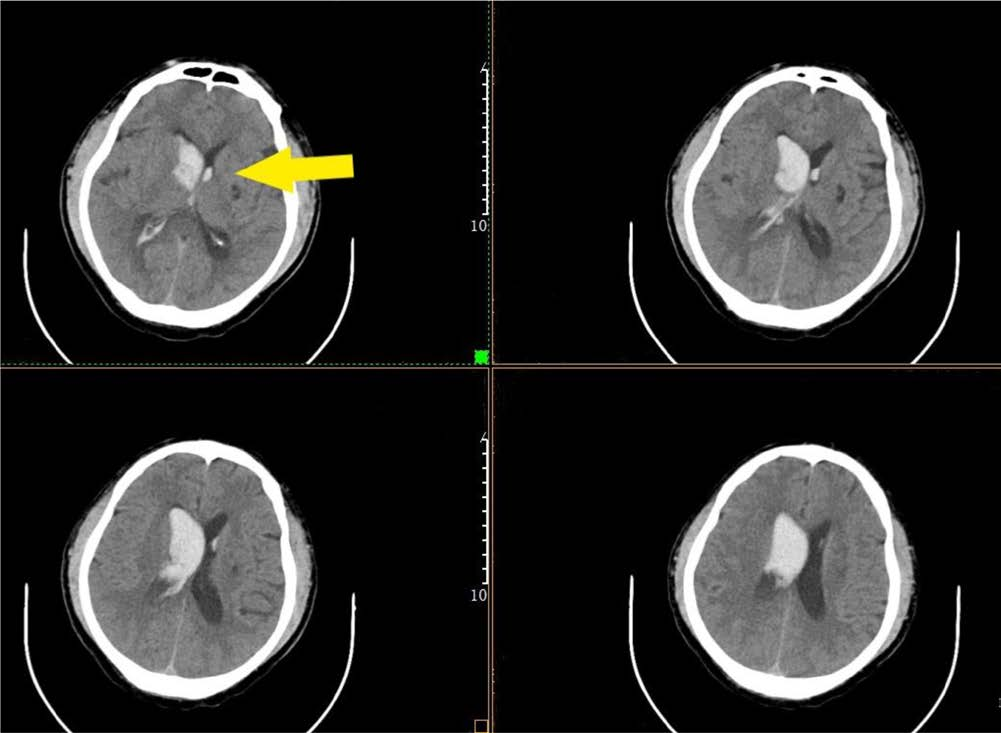
The right basal ganglia hemorrhage broke into the ventricle.

The patient presented with stroke-like symptoms (such as headache, nausea, and vomiting). Based on their physical examination, it is recommended to immediately use computed tomography (CT) or magnetic resonance imaging for rapid neuroimaging to confirm spontaneous cerebral hemorrhage. Therefore, head CT showed that the right basal ganglia hemorrhage had broken into the ventricle (Fig. 1). After consultation with neurosurgery, computed tomography angiography (CTA) was performed to rule out bleeding caused by intracranial vascular disease, and no abnormalities were found. The patient was admitted with “spontaneous cerebral hemorrhage.”

## 5. Differential diagnosis

Cerebrovascular malformation and cerebral aneurysm rupture bleeding, with bleeding symptoms similar to spontaneous cerebral hemorrhage, including headache, nausea and vomiting, or limb movement disorders; however, the bleeding site of cerebral vascular malformation is often not fixed, and the main bleeding site of cerebral aneurysm rupture is the subarachnoid hemorrhage, which can be distinguished by magnetic resonance angiography or digital subtraction angiography (DSA). The patient had a history of hypertension, and based on the bleeding site and age, it was considered that there was a high possibility of hypertension-related cerebral hemorrhage.

## 6. Treatment

**Figure 2.**
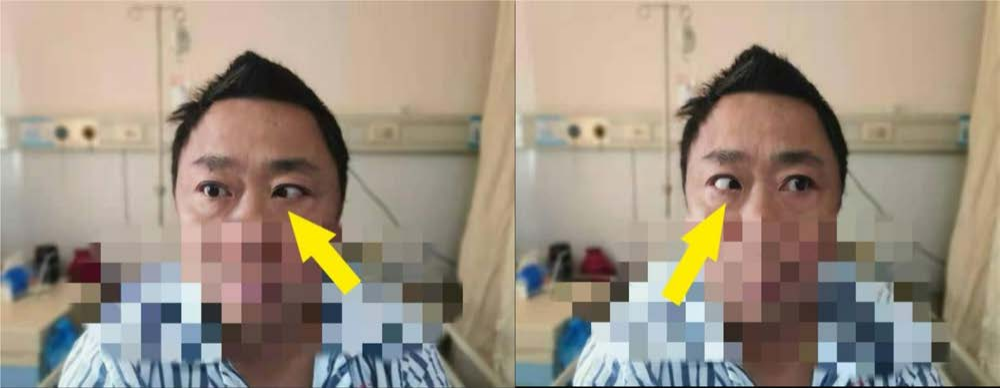
The patient had bilateral abduction limitation.

**Figure 3.**
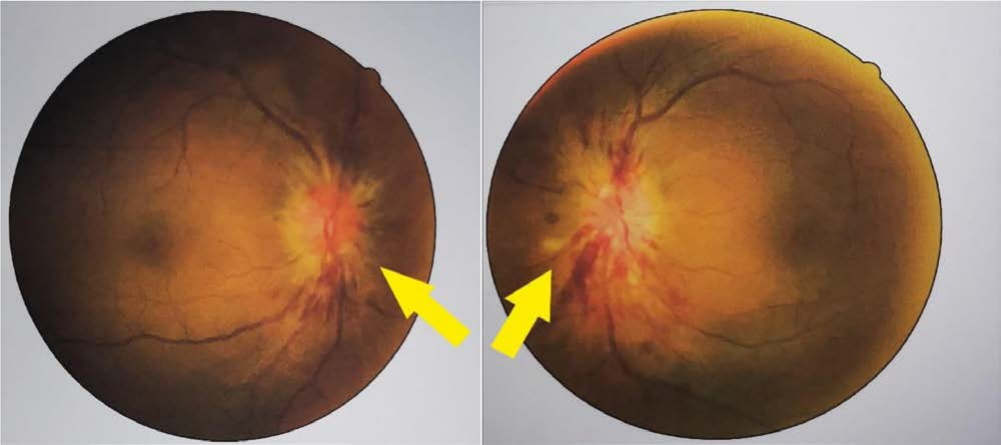
Hemorrhage and exudation of the optic papilla in both eyes, and the edema was obvious.

After admission, special nursing care, electrocardiogram monitoring, 3 L/min nasal catheter oxygen inhalation, and an indwelling catheter were administered. Relevant examinations improved, and symptomatic treatments such as hemostasis, nutritional rehydration, blood pressure control, and stable blood glucose were provided. CTA is a simple, rapid, and noninvasive method with high resolution. It has obvious timeliness advantages for the investigation of cerebrovascular diseases, but negative results cannot completely rule out the existence of lesions.^[20,21]^ DSA is the ‘ gold standard ‘ for examination of cerebrovascular diseases.^[1]^ Therefore, to more accurately rule out spontaneous cerebral hemorrhage caused by vascular causes, head CT showed that after the hematoma entered the absorption period, the patient underwent DSA under local anesthesia 2 weeks after admission. The results showed that: (1) there were no obvious abnormalities in the intracranial segment of the internal carotid arteries and the main trunk and main branches of the anterior and middle cerebral arteries on both sides. (2) There was no obvious abnormality in the main trunk or main branches of the vertebrobasilar artery on either side. Hypertensive cerebral hemorrhage was also considered. On the 4th day after DSA, the patient had bilateral abduction limitations (Fig. 2). CT reexamination showed that the intracranial hematoma had significantly improved compared with the previous absorption, and transient intracranial hypertension was considered. Ophthalmic consultation at the First People’s Hospital of Huzhou City showed hemorrhage and exudation of the optic papilla in both eyes, and edema was obvious (Fig. 3). Mannitol dehydration was used to reduce the intracranial pressure (ICP). The ICP was measured as to be 190 mm H₂O by lumbar puncture. Approximately 30 mL of cerebrospinal fluid was slowly collected, and the patients were administered neurotrophic drugs. It also improves the patient’s liver function and, provide patients with stable blood glucose and blood pressure, as well as other symptomatic support treatments.

## 7. Follow-up and outcomes

**Figure 4.**
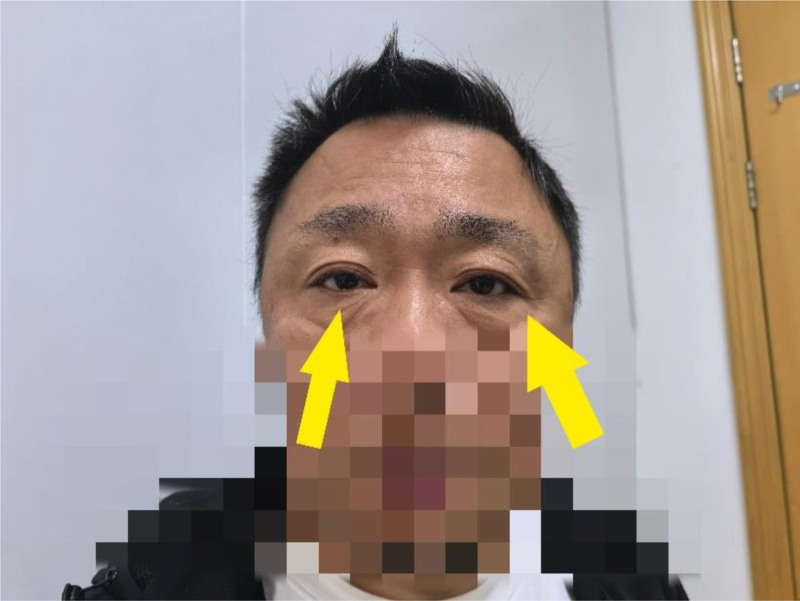
There was no abduction limitation in both eyes and the activity was good.

After treatment, the patient’s headache was better than before, and the left eye abduction limitation was better than before, but the right side was still not improved. One month after discharge, the patient was reexamined, and there was no abduction limitation in either eye and the activity was good (Fig. 4).

## 8. Discussion

This case presented with restricted bilateral abduction, which was suspected to be caused by a temporary increase in ICP after DSA due to hypertensive intraventricular hemorrhage. Spontaneous intracerebral hemorrhage can cause a series of pathological and physiological changes, usually manifested as rapid expansion of intracerebral hematoma, which can extend to the ventricular system, subarachnoid space, or subdural space, leading to an increased risk of recurrent cerebral hemorrhage, severe vascular events, epilepsy, dementia, and other neurological complications.^[22]^ Acute bleeding damages brain tissues and the nervous system, leading to brain dysfunction. However, a large hematoma can compress the surrounding brain tissue, increasing the pressure gradient between the hematoma area and the surrounding tissue and forcing the brain tissue to shift. Obstruction of the peripheral vascular circulation, cerebral ischemia, and hypoxia can lead to cerebral edema, resulting in a sharp increase in ICP, ultimately leading to cerebral herniation.^[23,24]^

When ICP increases, the pressure can not only directly act on the abducens nerve, but also move the brainstem downward through the pressure in the direction of the foramen magnum, thus involving the abducens nerve. It is pressed on the anterior inferior cerebellar artery or the apex of the rock bone, resulting in paralysis.^[25,26]^ The abducens nerve is pulled and compressed, resulting in symptoms such as diplopia and strabismus. If ICP is actively reduced in the early stages of the symptoms, symptoms such as nerve compression, traction, and diplopia can be improved.

The general treatment of ANP involves increasing the basic drug treatment based on the treatment of the primary disease, such as neurotrophic drugs, vasodilators, B vitamins, hormones, blood glucose regulation, and blood pressure control drugs.

The abducens nerve is the most vulnerable intracranial nerve, and whether it is adjacent to or far away from the brain damage can affect the abducens nerve, so simple ANP has no diagnostic value. The etiology of ANP is diverse and complex. This must be carefully and comprehensively examined, and systemic factors cannot be ignored. It is necessary to think more, analyze more, and ask carefully and comprehensively to understand the medical history to obtain more information for differential diagnosis. During clinical treatment, the cause of the disease should be carefully identified. In addition to understanding the medical history and symptoms, CT, magnetic resonance imaging, and other imaging methods should be used for screening.^[27]^ Detailed eye, nervous system, and laboratory examinations should be carefully performed.^[28]^ If necessary, relevant departments should consult and actively find the cause, so as to make a definite diagnosis and provide targeted treatment. If there are intracranial space-occupying lesions or aneurysms that may endanger life, care should be taken to avoid delaying the disease or improper treatment accidents. In a long-term clinical study, we found that the length of the course of the disease also has a great impact on the effect of treatment: the shorter the onset time, the earlier the intervention of the disease, and the better the effect will be. The improvement of the cure rate also has obvious help, so earlier detection, clear diagnosis, and active treatment of the disease are essential. In the presence of other primary diseases (such as hypertension and diabetes, etc), attention should be paid to the control of the primary disease. During the treatment period, vitamin B, vasodilators, and neurotrophic drugs can be added to achieve improved therapeutic effects.

## 9. Conclusion

Because the abducens nerve has a long shape at the base of the skull, it is the most vulnerable nerve innervating the extraocular muscle and is greatly affected by intracranial hypertension. Transient intracranial hypertension caused by DSA affects abducens nerve traction and causes paralysis.^[9,29]^ In the treatment of ANP, domestic and foreign scholars have conducted numerous experiments and research and made important contributions. In clinical treatment, the etiology and treatment of the primary disease should be clarified. At the same time, we should strengthen the research on the mechanism of the disease and the influence of different primary diseases on the treatment methods and results, and find quantitative criteria for the evaluation and observation of the disease. In clinical observation, we should pay more attention to the long-term treatment effect, and actively explore more efficient, safe, reliable, and easy-to-promote clinical treatment methods for the disease to contribute to the promotion of the treatment of ANP in the future.

## 10. Study limitations

The limitation of this study is that long-term follow-up was not conducted on the patient. After the initial follow-up, due to the patient’s cognitive limitations and inadequate health education provided by the hospital, long-term follow-up of the patient was not carried out. In future clinical work, it is necessary to conduct long-term follow-up on such patients in order to timely understand their condition.

## Author contributions

**Conceptualization:** Yong Cai.

**Supervision:** Xingming Zhong, Lijuan Shen.

**Writing – original draft:** Chenyan Li.

**Writing – review & editing:** Yong Cai.
